# Motor Simulation as an Adjunct to Patient Recovery Process Following Intensive Care Unit Admission

**DOI:** 10.3389/fmed.2022.868514

**Published:** 2022-03-17

**Authors:** Claire Calmels, Sébastien Le Garrec, Franck Brocherie

**Affiliations:** ^1^Laboratory Sport, Expertise and Performance (EA 7370), French Institute of Sport, Paris, France; ^2^Medical Department, French Institute of Sport, Paris, France

**Keywords:** intensive care medicine, immobilization, sensorimotor deprivation, motor learning, public health

## Introduction

Individuals commonly admitted to the intensive care unit (ICU) for acute respiratory failure, due to pulmonary or neuromuscular disease, shock and the need for airway protection or temporary respiratory support after major surgery, are generally placed on invasive mechanical ventilation support. The recovery processes of these patients is uncertain (the longer the duration of respiratory assistance, the more uncertain the vital prognosis) (1) and will take time even with the application of respiratory weaning process (i.e., spontaneous breathing periods alternated with respiratory assistance; enabling patients to gradually come back to unsupported spontaneous breathing) before leaving ICU (2). As these patients suffer from post-mechanical ventilation physical, cognitive, and mental sequelae, in addition to comorbidities (3), maximizing their recovery is therefore paramount to reduce any disabling experiences and to avoid the menace of relapse following discharge and consecutive risk of readmission (2).

It would be beneficial to adopt a multidisciplinary approach by pooling knowledge from the healthcare system—e.g., respiratory medicine and sport medicine—to advance the understanding of the (post-) ICU recovery process which will allow practitioners to set up the most beneficial rehabilitation program (4). In this view, based on neuroscience findings, we propose that Jeannerod's theory (5, 6) related motor simulation would be a useful complement to physiotherapy, motor rehabilitation and other accompanying interventions (e.g., speech therapy, nutritional, and psychological support) to optimize patients' return to autonomy.

## What Is Motor Simulation and Why Introducing Afferent Information During Motor Simulation?

**Figure 1:**
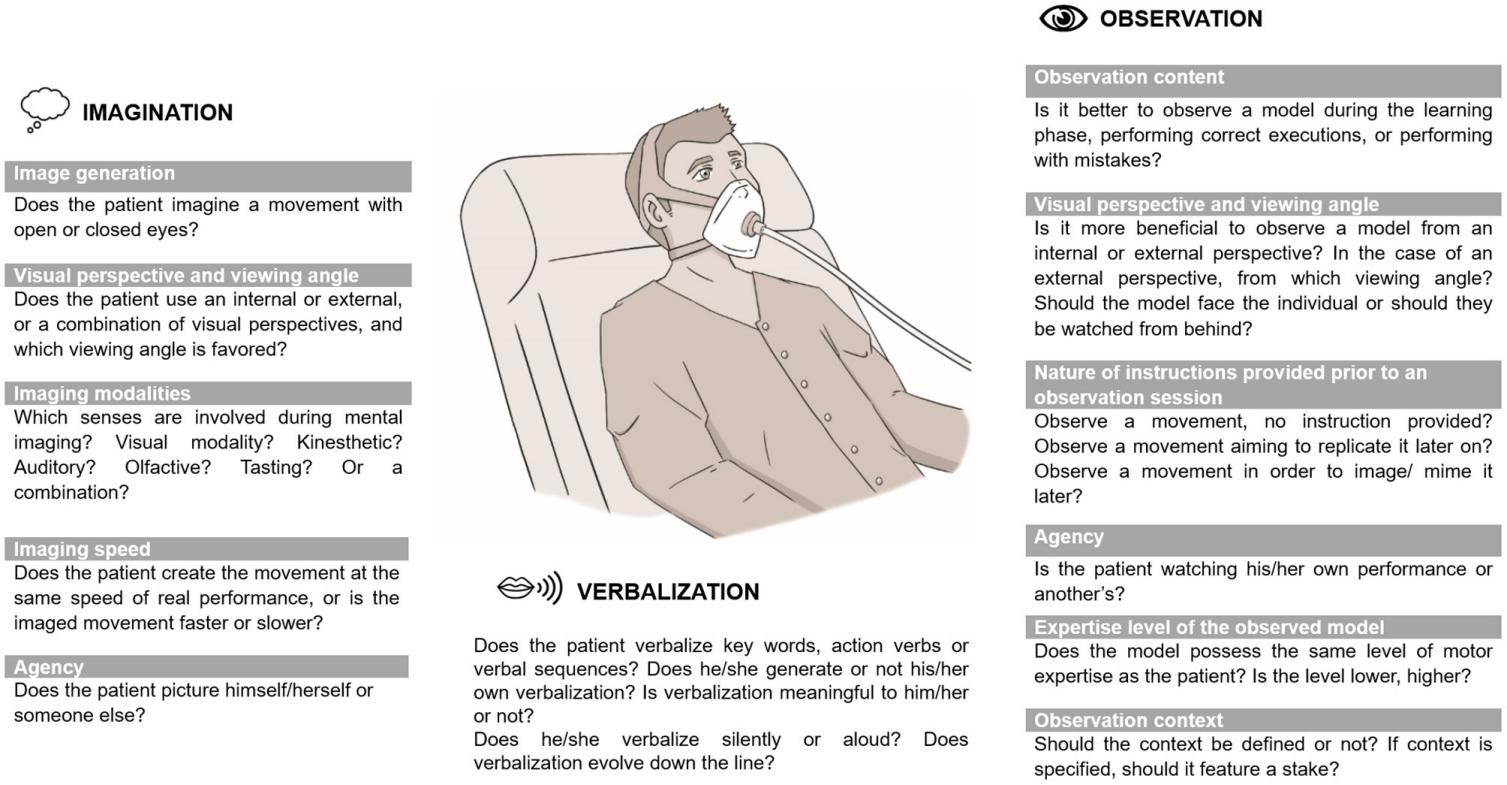
Principles to be considered and tailored to patients' individual needs for an efficient motor simulation process.

Ventilation support for 2–4 weeks and respiratory weaning care (i.e., alternating spontaneous breathing periods with respiratory assistance) result in significant muscle atrophy and weakness (3, 7). It imposes great challenge for optimal recovery from respiratory muscle fatigue due to patients' low breathing capacity, inability to perform basic movements and fatigability. Interestingly, motor simulation offers a motor learning/relearning alternative process without performing any movement (8). Briefly, an individual is engaged in motor simulation when he/she is able to imagine, observe or verbally describe a movement (Figure 1) (8). According to Jeannerod (5, 6), motor imagery or action observation (called covert stage) and action execution (called overt stage) share a common activation of cortical motor systems. Of interest, overt stages could be replayed off-line through motor simulation that enables the brain to represent the sensorial consequences and future states of simulated actions. In the field of neuroscience, systematic scientific reviews and meta-analysis have shown, first, that the imagination or the observation of a movement activates a premotor-parietal and primary somatosensory network similarly to the one involved during “real” movement execution (9); second, that premotor and primary motor areas are activated during arm or leg movement verbalization and execution (10); third and last, as suggested almost two decades ago (11), that human brain is provided by internal motor efference copies between simulated (imagined, observed, verbalized) and “real” movements (12–14). While simulating or performing movements, these internal forward models are enrolled to predict movement-induced future states and sensorial consequences. Thus, simulated movements are movements except for the fact they do not generate motor output which is blocked by a motor command inhibitory mechanism (5, 6).

However, during a motor simulation, the absence of sensorimotor feedback (i.e., sensorimotor consequences of the motor command) could inactivate the somatosensory processes underlying movement representation, thereby restricting the impact of motor simulation on motor learning/relearning process (15). In the same vein, others have argued that peripheral (proprioceptive and haptic) information is essential for accessing and maintaining motor representations stored in the brain and that, in their absence, these representations fade and even cease to be accessible (16). For instance, the effects of transient sensorimotor deprivation following orthopedic trauma in patients with no prior neurological deficits revealed motor execution impairments and maladaptive plasticity in the somatosensory and motor cortices including shrinkage of somatosensory cortical maps, decreased cortical excitability, reduced cortical thickness in the sensorimotor cortex, decreased of fractional anisotropy in the corticospinal tract and decreased interhemispheric inhibition from the impacted to the non-impacted hemisphere (8). The impaired functioning of internal forward models (referring to the interactions between motor commands and environment, useful to predict the sensory consequences of an action) accounts for these alterations. Though, these models need to be updated through experience (i.e., training that can be translated into changes in synaptic weights which will improve future forward model prediction) to operate reliably (11). Because of the lack of sensorimotor input (proprioceptive, haptic) in the case of immobilization for instance, forward models remain available but are no longer updated, generating an inaccurate prediction of sensory consequences, and thus initiating unskilled and inefficient movements (11). Consequently, integrating afferent information (i.e., proprioceptive and haptic) during motor simulation should be encouraged (i.e., mimicking a movement and handling an object related to a specific movement) (8). It has been reported that mimicking a movement concomitantly to its motor/mental simulation increases its technical quality and efficacy (17).

## What Is the Evidence For a Positive Impact of Motor Simulation In Real-World Clinical Practice?

Studies probing the effects of motor simulation interventions prescribed alongside motor rehabilitation to recover from detrimental effects of transient sensorimotor deprivation are scarce in individuals without any neurological history (8). Notably, in patients over 60 years who underwent total hip and knee arthroplasty surgery were instructed to feel the sensations of movements they mentally simulated, results indicated some benefits through an increased motor performance following observation of locomotor tasks (18) and greater quadriceps strength subsequent to the imagination of knee flexion-extension (19). These positive outcomes are deemed to be due to equivalences between simulated and executed movements (18, 19), to the integration of proprioceptive information during motor simulation (8, 17), to the combination of motor rehabilitation and simulation sessions (18, 19), and to cortical plasticity in response to internal or external constraints such as motor learning or training interventions (8, 18, 19). The scientific community hence agrees that cortical changes in healthy individuals exposed to motor learning/training are seen in brain areas and networks related to the physical execution of movements.

## Why Using Motor Simulation for ICU and Post-ICU Patients?

Despite the lack of scientific evidence promoting motor simulation benefits to ICU and/or post-ICU patients so far, its integration in the rehabilitation process could appear as a very promising adjunct for the following reasons.

First, delivering early active mobilization and rehabilitation in ICU, when the cardiovascular, respiratory and neurological states of patients are stable (20), has been shown to improve the rehabilitation outcomes (improved muscle strength, functional capacity and mobility; increased number of ventilator-free day and discharged-to-home rate) (21). In this view, motor simulation can be helpful in the early rehabilitation process (i.e., when the patient is temporarily completely or partially unable to move, too weak to exercise or subject to the use of supportive devices), as it offers a motor learning/relearning alternative without requiring patient to perform any movement (8). Whenever possible, it is important to alternate motor simulation and movement execution, whether active or assisted, within the same session, as the benefit of this approach has been demonstrated (18, 19).

Second, because ICU patients suffer from sensory deprivation increased by the interference of sedative and analgesic medication, it is essential to provide visual, proprioceptive, haptic information during motor simulation (e.g., watching a video of a movement, mimicking it, handling an object related to this movement). Interestingly, as observing an object automatically potentiates actions associated to it (22), showing objects that patient used in their daily lives will activate action representations (e.g., reaching or grasping actions when a mug is shown).

Third, depending on an individual's ability to use motor simulation techniques (e.g., patients with poor imagery ability), on his/her fatigability, motivation and individual needs, a variety of different simulation packages could be offered for an optimal motor simulation process. The Figure 1 summarizes the available motor simulation techniques that can be used alone or in combination (i.e., preferably associated with miming a movement and/or handling an object related to this movement to generate afferent information during (motor) simulation) and declined under varied modalities (8) to personalize the intervention in reference to the patient's requirements.

## Concluding Remarks

The present opinion aimed to promote motor simulation as a plausible non-invasive, safe, easy to implement and low cost complementary adjunct among the healthcare delivery provided during the (post-) ICU recovery process. Hopefully, the ICU practitioners and their multidisciplinary healthcare professionals will consider motor simulation as a practical, relevant and therapeutic option to maximize patient's return to autonomy.

## Author Contributions

The authors listed have made substantial, direct, and intellectual contribution to the work and approved it for publication.

## Conflict of Interest

The authors declare that the research was conducted in the absence of any commercial or financial relationships that could be construed as a potential conflict of interest.

## Publisher's Note

All claims expressed in this article are solely those of the authors and do not necessarily represent those of their affiliated organizations, or those of the publisher, the editors and the reviewers. Any product that may be evaluated in this article, or claim that may be made by its manufacturer, is not guaranteed or endorsed by the publisher.

## References

[1] Sun Y Li S Wang S Li C Li G Xu J. Predictors of 1-year mortality in patients on prolonged mechanical ventilation after surgery in intensive care unit: a multicenter, retrospective cohort study. BMC Anesthesiol. (2020) 20:44. 10.1186/s12871-020-0942-0 32085744 PMC7033944

[2] Vincent JL. The post-ICU patient. ICU Manag Pract. (2020) 20:238. Available online at: https://healthmanagement.org/c/icu/issuearticle/the-post-icu-patient-1

[3] Haviland K Tan KS Schwenk N Pillai MV Stover DE Downey RJ. Outcomes after long-term mechanical ventilation of cancer patients. BMC Palliat Care. (2020) 19:42. 10.1186/s12904-020-00544-x 32228554 PMC7106688

[4] Faghy MA Ashton RE Maden-Wilkinson TM Copeland RJ Bewick T Smith A. Integrated sports and respiratory medicine in the aftermath of COVID-19. Lancet Respir Med. (2020) 8:852. 10.1016/S2213-2600(20)30307-6 32653073 PMC7347343

[5] Jeannerod M. Neural simulation of action: a unifying mechanism for motor cognition. Neuroimage. (2001) 14:S103–9. 10.1006/nimg.2001.0832 11373140

[6] Jeannerod M. The representing brain: neural correlates of motor intention and imagery. Behav Brain Sci. (1994) 17:187–202. 10.1017/S0140525X00034026

[7] MacIntyre NR Epstein SK Carson S Scheinhorn D Christopher K Muldoon S. Management of patients requiring prolonged mechanical ventilation: report of a NAMDRC consensus conference. Chest. (2005) 128:3937–54. 10.1378/chest.128.6.3937 16354866

[8] Calmels C. Beyond Jeannerod's motor simulation theory: an approach for improving post-traumatic motor rehabilitation. Neurophysiol Clin. (2019) 49:99–107. 10.1016/j.neucli.2019.01.033 30685210

[9] Hardwick RM Caspers S Eickhoff SB Swinnen SP. Neural correlates of action: comparing meta-analyses of imagery, observation, and execution. Neurosci Biobehav Rev. (2018) 94:31–44. 10.1016/j.neubiorev.2018.08.003 30098990

[10] Hauk O Johnsrude I Pulvermuller F. Somatotopic representation of action words in human motor and premotor cortex. Neuron. (2004) 41:301–7. 10.1016/S0896-6273(03)00838-9 14741110

[11] Wolpert DM Flanagan JR. Motor prediction. Curr Biol. (2001) 11:R729–32. 10.1016/S0960-9822(01)00432-8 11566114

[12] Gazzola V Keysers C. The observation and execution of actions share motor and somatosensory voxels in all tested subjects: single-subject analyses of unsmoothed fMRI data. Cereb Cortex. (2009) 19:1239–55. 10.1093/cercor/bhn181 19020203 PMC2677653

[13] Whitford TJ Jack BN Pearson D Griffiths O Luque D Harris AW. Neurophysiological evidence of efference copies to inner speech. Elife. (2017) 6:e28197. 10.7554/eLife.28197 29199947 PMC5714499

[14] Kilteni K Andersson BJ Houborg C Ehrsson HH. Motor imagery involves predicting the sensory consequences of the imagined movement. Nat Commun. (2018) 9:1617. 10.1038/s41467-018-03989-0 29691389 PMC5915435

[15] Lacourse MG Turner JA Randolph-Orr E Schandler SL Cohen MJ. Cerebral and cerebellar sensorimotor plasticity following motor imagery-based mental practice of a sequential movement. J Rehabil Res Dev. (2004) 41:505–24. 10.1682/JRRD.2004.04.0505 15558380

[16] Bosbach S Cole J Prinz W Knoblich G. Inferring another's expectation from action: the role of peripheral sensation. Nat Neurosci. (2005) 8:1295–7. 10.1038/nn1535 16136040

[17] Guillot A Moschberger K Collet C. Coupling movement with imagery as a new perspective for motor imagery practice. Behav Brain Funct. (2013) 9:8. 10.1186/1744-9081-9-8 23425312 PMC3599464

[18] Marusic U Grospretre S Paravlic A Kovac S Pisot R Taube W. Motor imagery during action observation of locomotor tasks improves rehabilitation outcome in older adults after total hip arthroplasty. Neural Plast. (2018) 2018:5651391. 10.1155/2018/5651391 29755513 PMC5884021

[19] Moukarzel M Di Rienzo F Lahoud JC Hoyek F Collet C Guillot A. The therapeutic role of motor imagery during the acute phase after total knee arthroplasty: a pilot study. Disabil Rehabil. (2019) 41:926–33. 10.1080/09638288.2017.1419289 29275638

[20] Devlin JW Skrobik Y Gelinas C Needham DM Slooter AJC Pandharipande PP. Clinical practice guidelines for the prevention and management of pain, agitation/sedation, delirium, immobility, and sleep disruption in adult patients in the ICU. Crit Care Med. (2018) 46:e825–e73. 10.1097/CCM.0000000000003299 30113379

[21] Zhang L Hu W Cai Z Liu J Wu J Deng Y. Early mobilization of critically ill patients in the intensive care unit: a systematic review and meta-analysis. PLoS ONE. (2019) 14:e0223185. 10.1371/journal.pone.0223185 31581205 PMC6776357

[22] Grezes J Tucker M Armony J Ellis R Passingham RE. Objects automatically potentiate action: an fMRI study of implicit processing. Eur J Neurosci. (2003) 17:2735–40. 10.1046/j.1460-9568.2003.02695.x 12823480

